# Supplementary material for: Hepcidin Upregulation in Colorectal Cancer Associates with Accumulation of Regulatory Macrophages and Epithelial–Mesenchymal Transition and Correlates with Progression of the Disease
Source: Cancers (Basel). 2022 Oct 27;14(21):5294. doi: 10.3390/cancers14215294 (PMC9658525; doi:10.3390/cancers14215294)
Supplement: Supplementary file 1 [file cancers-14-05294-s001.zip › cancers-1947006-supplementary.pdf]

cancers

MDPI

Supplementary Figure S1

a

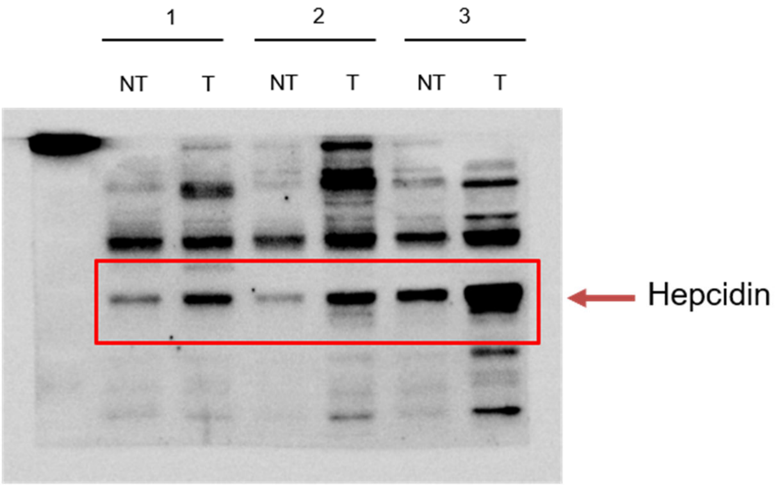

b

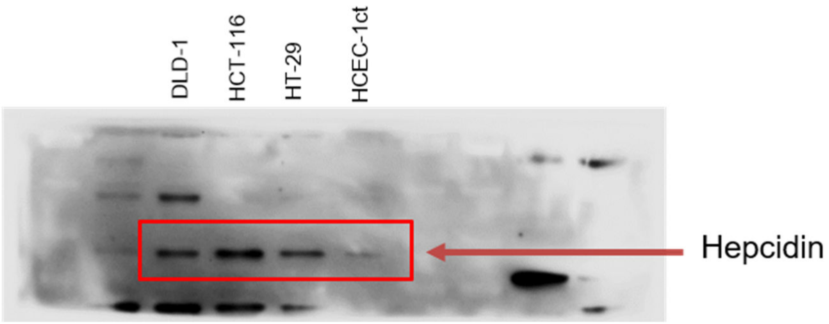

Figure S1. Uncropped western blots to hepcidin expression. a. Figure 1a. b. Figure 1d.

Supplemantary Figure S2

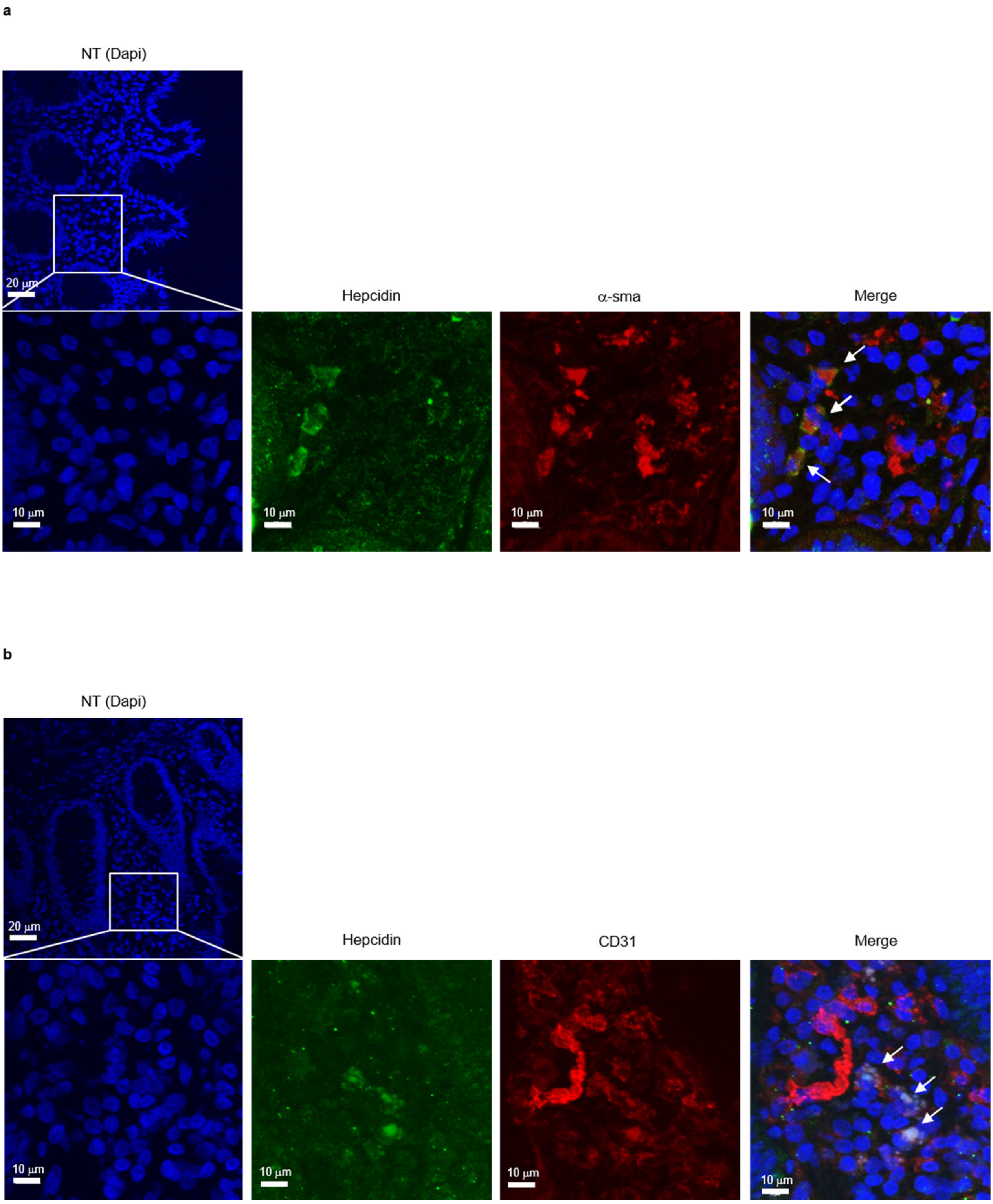

Figure S2. Representative confocal laser scanning microscopy images. a. α-SMA (red) and hepcidin (green) in uninvolved, non-tumoral mucosa (NT) of a CRC patient; nuclei are stained with 4′,6-diamidino-2-phenylindole (DAPI) (blue). White arrows indicate cells co-expressing α-SMA and hepcidin. b. CD31 (red) and hepcidin (green) in uninvolved non-tumoral mucosa (NT) of a CRC patient; nuclei are stained with 4′,6-diamidino-2- phenylindole (DAPI) (blue). White arrows indicate cells co-expressing CD31 and hepcidin.

Supplementary Figure S3

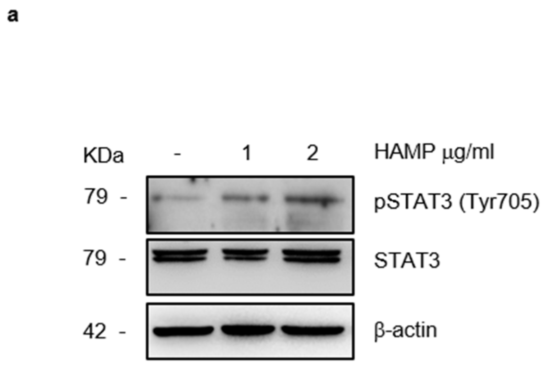

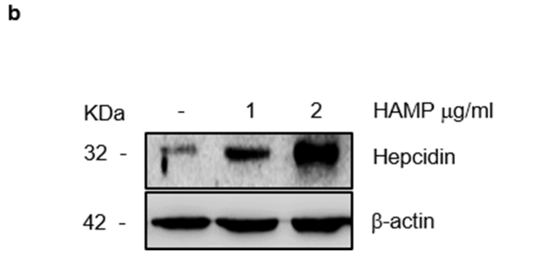

Figure S3. a. Representative Western blots showing p-Stat3, Stat3 and β-actin in HCT-116 cells stimulated with recombinant hepcidin for 30′ minutes. b. Representative Western blots showing hepcidin and β-actin in HCT-116 cells stimulated with recombinant hepcidin for 24 hours.

Figure supplementary S4

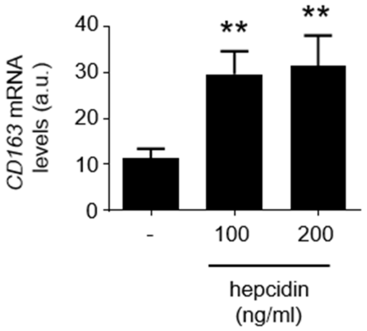

Figure S4. Stimulation of blood mononuclear cells with hepcidin increases CD163, a marker of regulatory macrophages. Blood mononuclear cells were either left unstimulated (Unst) or stimulated with exogenous hepcidin as indicated in materials. CD163 RNA expression was analyzed by Real-Time PCR and values were normalized to β-actin RNA. Values are expressed in arbitrary units (a.u.) and indicate mean ± SD of 3 experiments. **$p<0.01$.
